# Love writing

**DOI:** 10.7554/eLife.45734

**Published:** 2019-03-07

**Authors:** Eve Marder

**Affiliations:** 1 Volen CenterBrandeis UniversityWalthamUnited States; 2 Biology DepartmentBrandeis UniversityWalthamUnited States

**Keywords:** Living Science, careers in science, scientific publishing, early-career researchers, peer review, writing

## Abstract

Clear writing is the key to success.

In my early years I always thought of myself as an intellectual, and like my mother, I was a snob. As a teenager I read the classics and listened to Bach, Beethoven, and Mozart (along with Bob Dylan and Aretha Franklin, of course). I visited art museums and sought out mainstream and avant garde films and plays. At age 22 I would have been appalled to know that, many years later, I would follow professional sports and take life lessons from the commentators on sports radio (whom I often listen to in the car to hide from the pain of the actual news). Maybe this isn’t entirely surprising because I played tennis, volleyball, basketball, and soccer for many years.

One of the most common discussion points on sports radio is the difference in attitude and behavior shown by rookies (young first-year players) and the seasoned veterans (who were once themselves rookies). It is commonly accepted that a healthy and productive team needs to take advantage of the strength, enthusiasm, and agility of its youngest members. Nonetheless, the commentators often discuss the necessity that the new players learn from the veterans, and they are convinced that the successful teams are those that profit from the seamless combination of exuberance and experience. Moreover, the long-term success of a team derives in large part from its ability to motivate the veterans to mentor the younger players, and to signal to the rookies that they have much to learn from their older team mates. Sustained excellence requires generational transfer of knowledge and best practices, both in sports and science.

The future of our community rests with the success of our graduate students, postdocs, and beginning faculty members. It is disconcerting to discover, therefore, that many of our brightest and most innovative young scientists struggle with their first papers and first grant applications, and will often, almost proudly, say that they hate writing. I don’t mind knowing that they find writing difficult (it certainly can be!), but no active and creative scientist can afford to hate writing, because our published work is the currency of our profession.

Data show that early-career scientists are at a disadvantage in getting grants and in publishing their first papers. Some posit that early-career scientists are disadvantaged because they are less well known than more senior scientists. Although, that is certainly part of the answer, many of the difficulties that early-career scientists face come from what more seasoned scientists recognize as 'rookie errors' because we have seen them over and over in first papers and first grant proposals.

An example: many years ago I was on an NIH Study Section and was assigned a grant from a new investigator. It seemed at first glance to suggest way too much work (being too ambitious is the most common failing of first grant applications). In this case, I estimated that it would take approximately 250 person years to do the experiments proposed for four people over a five year grant period. Why was this such a blunder? It was lethal for the proposal because it signaled to the reviewer that the principal investigator had not carefully thought through what it would take to do the work. Or even worse, he or she did not intend to do the work carefully and completely. Obviously, most successful grant applications propose more than can be done in the time allotted: however, being ambitious by a factor of two is within expected norms, whereas being ambitious by a factor of ten or more only erodes the credibility of the proposer. And while a grant panel might be willing to give a senior investigator a break by saying their past track record indicates that they will be able to overcome unforeseen obstacles, if a new investigator loses credibility by proposing vastly too many experiments, the panel will be unable to judge what he or she actually intends to do, and will consequently score the application poorly.

**Figure 1:**
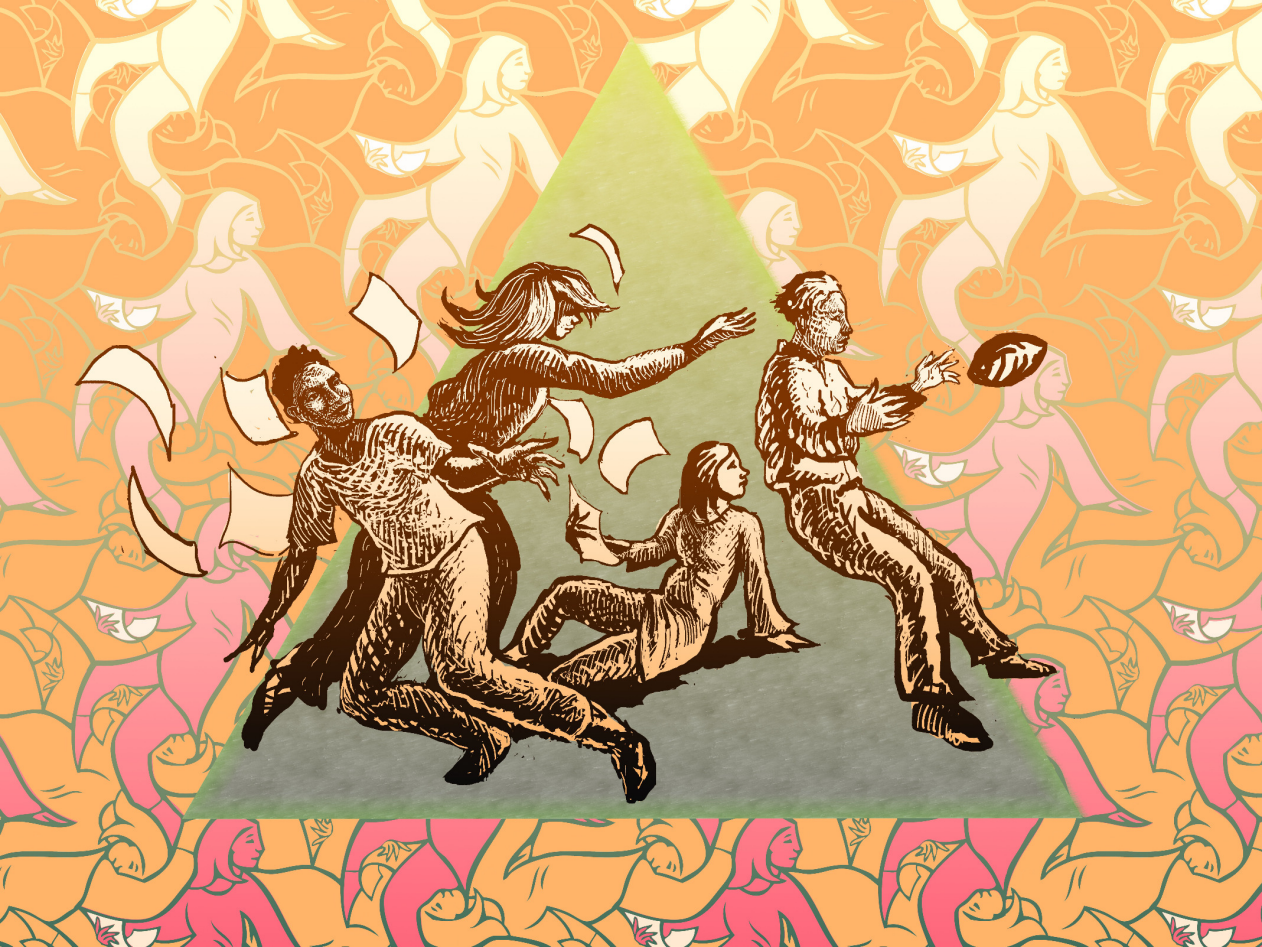
Sports teams recognize the importance of young players ('rookies') and seasoned veterans working together, and the same is true in science.

Illustration: Ben Marder.

Similar issues arise during manuscript review. Many manuscripts are plagued by errors of 'overselling', in which authors enthusiastically argue that their papers are paradigm shifting and will change the entire trajectory of science in a given field. Needless to say, this is rarely the case. Sadly, early-career scientists are often taught by their experienced mentors that overselling is necessary to get a paper accepted by a prestige journal. In reality, at eLife the most common complaint of reviewers of manuscripts from authors at all career stages is that the manuscript is 'oversold'. Reviewers are often content with authors just describing what they did and why. That said, there is a fine line between overselling and articulating clearly the new messages of the work. We all struggle with this as we try to frame our work for the reader. It is very hard to position a paper honestly so that its new contribution is evident, without crossing the line into 'overselling'.

While some postdocs do write their own papers, many are part of large groups and the paper emerges from ensembles of authors within a framework devised by one or more senior authors. This experience may not provide much training for the postdoc when approaching his or her own first papers from their independent laboratory.

At *eLife* we often see papers from early-career scientists that suffer because the authors assume too much from the reader, or because the paper does not do justice to the intellectual framework that motivated the work. And we see papers that are missing appropriate controls, or papers in which the authors seem to be confused about what they are trying to say or what they can conclude from their experiments: I should stress that the latter problem is not restricted to early-career authors. I have found that my most interesting and important papers were some of the most difficult to write, precisely because we were struggling to understand the implications and logic of the findings. In many cases, I have understood the work through the act of writing about it, even sometimes rewriting after an initial version was rejected.

A large fraction of submitted papers are criticized for being poorly written or unclear. But, as in so many things, practice and experience do help. I have my own simple suggestions that can help an author to write a clear paper. 1) Start writing the paper before the experiments are 100% complete, as often a missing piece only becomes apparent when you are 'telling the story'. 2) Write the first draft quickly so that you remember what the beginning says as you work on the middle and end of the manuscript. It is very hard to get the rhythm of a piece right if you put it down for too long. If you write the Introduction in a few hours and realize it isn’t working, it is much easier to throw it away and start over than if you have spent two weeks on it. 3) Be prepared for lots of drafts. Some papers can be completed in a few drafts, but others require many more. As part of this, you have to be prepared to delete the perfect sentence or paragraph when it isn’t serving its function. 4) When you think you have a semi-final draft, go through it and get rid of excess words, sentences, and paragraphs. Read the text aloud to yourself, as your ears are more likely to pick up awkward syntax than your eyes. 5) Find a ruler and measure the number of inches or centimeters that you are dedicating to the different points in the paper, and make sure that the paper’s real message is not buried in pages of less interesting details or caveats.

But most importantly, writing is the medium that allows you to explain, for all time, your new discoveries. It should not be a chore, but an opportunity to share your excitement, and maybe your befuddlement. It allows each of us to add to and modify the conceptual frameworks that guide the way we understand our science and the world. Without papers, the data are unanchored. And surely, one of the great pleasures of doing science is seeing your work change the way others think about an interesting and previously mysterious question. It is not an accident that some of our best and most influential scientists write elegant and well-crafted papers. So, work to make writing one of the great pleasures of your life as a scientist, and your science will benefit.

